# Using Rasch rating scale model to reassess the psychometric properties of the Persian version of the PedsQL^TM ^4.0 Generic Core Scales in school children

**DOI:** 10.1186/1477-7525-10-27

**Published:** 2012-03-13

**Authors:** Peyman Jafari, Zahra Bagheri, Seyyed Mohamad Taghi Ayatollahi, Zahra Soltani

**Affiliations:** 1 Department of Biostatistics, Shiraz University of Medical Sciences, Shiraz, Iran

**Keywords:** quality of life, school children, Iran, Rasch model

## Abstract

**Background:**

Item response theory (IRT) is extensively used to develop adaptive instruments of health-related quality of life (HRQoL). However, each IRT model has its own function to estimate item and category parameters, and hence different results may be found using the same response categories with different IRT models. The present study used the Rasch rating scale model (RSM) to examine and reassess the psychometric properties of the Persian version of the PedsQL^TM ^4.0 Generic Core Scales.

**Methods:**

The PedsQL^TM ^4.0 Generic Core Scales was completed by 938 Iranian school children and their parents. Convergent, discriminant and construct validity of the instrument were assessed by classical test theory (CTT). The RSM was applied to investigate person and item reliability, item statistics and ordering of response categories.

**Results:**

The CTT method showed that the scaling success rate for convergent and discriminant validity were 100% in all domains with the exception of physical health in the child self-report. Moreover, confirmatory factor analysis supported a four-factor model similar to its original version. The RSM showed that 22 out of 23 items had acceptable infit and outfit statistics (<1.4, >0.6), person reliabilities were low, item reliabilities were high, and item difficulty ranged from -1.01 to 0.71 and -0.68 to 0.43 for child self-report and parent proxy-report, respectively. Also the RSM showed that successive response categories for all items were not located in the expected order.

**Conclusions:**

This study revealed that, in all domains, the five response categories did not perform adequately. It is not known whether this problem is a function of the meaning of the response choices in the Persian language or an artifact of a mostly healthy population that did not use the full range of the response categories. The response categories should be evaluated in further validation studies, especially in large samples of chronically ill patients.

## Background

There are two different methods for testing psychometric properties of quality of life (QoL) instruments, including classical test theory (CTT) and item response theory (IRT). Despite the popularity of CTT, it is unable to consider important aspects of measurement such as item difficulty, item discrimination and ordering of response categories [1]. Traditional psychometric techniques, called CTT, focus on summated scores, in which the scores on multiple items are added together [2]. In IRT, unlike in CTT, the properties of items can be analyzed individually with respect to the amount of information they provide about the latent trait [3]. Most QoL instruments have items that are rated on a Likert scale. Hence, there are a number of ordered polytomous IRT models, such as the graded response model (GRM), the partial credit model (PCM), the rating scale model (RSM), and the generalized partial credit model (GPCM), all of which have been used in QoL research. Despite their advantages, IRT models are sometimes difficult to apply in quality of life studies. These models require two crucial assumptions including unidimensionality and local independence to estimate the model parameters. Moreover, they need a huge sample size for acceptable performance [4,5]. In recent years, using IRT models has extensively increased in measuring HRQoL in school children [1,3,6-16]. The PedsQL^TM ^4.0 Generic Core Scales (hereinafter referred to as PedsQL^TM ^4.0) is one of the most popular instruments which measures HRQoL in both healthy and chronically ill children. The psychometric properties of the PedsQL^TM ^4.0 have been previously evaluated according to CTT [17-22]. For instance, its factorial structure has been assessed by exploratory or confirmatory factor analysis. Also, convergent and discriminant validity have been evaluated by Pearson or Spearman correlation. Although the results have been satisfactory, CTT has not considered item properties and person characteristics in the model. Moreover, CTT has not been able to determine whether or not respondents use the categories of a rating scale in the fashion intended by test developers. To the best of our knowledge, there are three published studies that have evaluated the psychometric properties of the PedsQL^TM ^4.0 using IRT models [23-25]. Kook and Varni [23] have provided a comprehensive review of the use of IRT and CTT in the validation of the Korean version of the PedsQL^TM ^4.0. Hill et al. [24] have demonstrated the value of the categorical confirmatory factor analysis to test the IRT model assumptions, including local dependence and unidimensionality. Moreover, Langer et al. [25] have used differential item functioning analyses (DIF) to assess whether scores have equivalent meaning across healthy children and children with chronic conditions.

Although the feasibility, reliability and validity of the Persian version of the PedsQL^TM ^4.0 have been recently approved among school children [26] and in children with chronic diseases [27,28], its psychometric properties are still unknown in some cases. Therefore, this study aims to test the psychometric properties of the Persian version of the PedsQL^TM ^4.0 using ordered polytomous IRT to determine whether this model provides information that cannot be obtained from CTT.

## Material and methods

### Participants and instrument

The Persian version of the PedsQL^TM ^4.0, which had been translated and validated previously in Iran [26,27], was completed by 938 school children aged 8-18 years and their parents. The participants were randomly selected by a two-stage cluster random sampling technique from the four educational districts of Shiraz, southern Iran. The 23-item PedsQL^TM ^4.0 consists of four domains including physical health (8 items), emotional functioning (5 items), social functioning (5 items), and school functioning (5 items). Items were scored on a 5-point Likert response scale (0 = never a problem, 1 = almost never a problem, 2 = sometimes a problem, 3 = often a problem, and 4 = almost always a problem). The numerical scale from 0 to 4 was included in the Persian translation of the PedsQL^TM ^4.0 questionnaire as well as the verbal descriptions. All the domains were transformed to a 0-100 scale (0 = 100, 1 = 75, 2 = 50, 3 = 25, 4 = 0), so that higher scores indicated better HRQoL.

## Statistical analysis

### CTT analysis

Reliability and validity of the PedsQL^TM ^4.0 were assessed using the traditional CTT approach. Internal consistency was assessed by the Cronbach's alpha coefficient for each domain. It was considered satisfactory if the coefficient was equal or greater than 0.7. Convergent and discriminant validity were evaluated using Spearman correlation. The value of a correlation coefficient of greater than 0.40 between an item and its own domain was regarded as an adequate evidence of convergent validity. Discriminant validity was supported whenever a correlation between an item and its hypothesized domain was higher than its correlation with the other scales. If the item to own-scale correlation was significantly higher than the correlations of the item to other domain, it was considered as scaling success [2]. Construct validity was assessed by the categorical confirmatory factor analysis (CCFA).

### IRT analysis

The CCFA was used to check IRT model assumptions including unidimensionality and local independence. Local independence means that all pairs of items within a domain should be uncorrelated after controlling for the latent trait. If the assumption of unidimensionality holds, a one-factor model should fit the data in each domain [2,4,5]. Goodness of fit was investigated based on root mean square error of approximation (RMSEA), non-normed fit index (NNFI), comparative fit index (CFI), and root mean square residual (RMR). Values of RMSEA less than 0.05 indicate close fit, less than 0.08 a reasonable fit and greater than 0.1 a poor fit [29]. Values of NNFI greater than 0.92 [29] and CFI greater than 0.9 are considered as a good fit and values of RMR close to 0 show an acceptable fit [30]. In addition, large modification indices for the error covariances indicate local dependence. The size of a modification index should be considered with respect to other modification indices and also the magnitude of the chi-square statistic [24]. The LISREL 8.54 software was used to perform the CCFA.

The RSM was used to assess person and item reliability, item statistics and ordering of response categories. Parameters for this model were estimated using the program WINSTEP [31]. The RSM assumes all items are equally discriminating and have the same number of response categories [4,5].

The probability of responding in category g of item i for the RSM, contingent on θ, is:

$${\text{P}}_{\text{ig}}=\frac{\text{exp}\overset{1}{\underset{\text{g}=0}{\sum }}\left[\theta -\left({\text{b}}_{i}+\tau_{\text{g}}\right)\right]}{\overset{\text{m}}{\underset{\text{h}=0}{\sum }}\left[\text{exp}\overset{\text{h}}{\underset{\text{g}=0}{\sum }}\left[\theta -\left({\text{b}}_{\text{i}}-\tau_{\text{g}}\right)\right]\right]},$$

where h = 0,1,..., g,..., m and g represents a specific category which is modeled; there are *m + *1 response categories in the item; θ represents the continuous latent trait (person location); and *b*_*i *_is the difficulty (location) parameter for item *i *and τ_*g *_is step (category) measure. Due to sensible interpretation of the RSM parameters, we reversed rating scale categories such that higher scores represented higher quality of life (4 = never, 3 = always never, 2 = sometimes, 1 = often, and 0 = almost always).

Two fit indices including the infit and outfit mean square (MNSQ) statistics were used to investigate whether all items contributed adequately to their own domain. Infit MNSQ is an information-weighted mean square residual which is more sensitive to unexpected response of persons whose abilities are near item difficulty, while outfit is unweighted mean square residual being more sensitive to unexpected outlying observations [4]. A MNSQ value greater than 1.4 indicates that the item fails to define the same construct as the other items do in a domain. MNSQ values lower than 0.6 may be an indication of item redundancy and values about 1.0 are ideal [23].

Moreover, the RSM was also used in order to identify whether successive response categories for each item are located in the expected order. Step measure, average measure and category fit statistics as well as category probability curves were used as diagnostic tools for assessing category functioning [32]. The step measure parameter defines the boundaries between categories which should increase monotonically with categories. Disordering of step measures occurs when the rating scale does not function properly [33]. Average measure is the average of the ability estimates for all participants who choose a particular category, which is expected to advance monotonically with categories [32,33]. Category fit was evaluated by the infit and outfit mean square statistic computed for each rating category. The categories were considered as misfitting if infit or outfit statistics were >1.4 or <0.6 [23]. Furthermore, the RSM provides item and person separation for assessing questionnaire functioning. Regarding the underlying construct, the person separation index represents how well persons can be discriminated by the questionnaire and the item separation index represents how well the items can be separated by the questionnaire [34]. The acceptable value of separation indices is 2.0, which leads to a value of >0.8 for the corresponding person and item reliabilities [35].

The Rasch RSM also visually inspected targeting of item difficulty to person ability by the person-item map showing persons and items on the same logit scale. Optimal targeting occurs when a set of items in a domain are able to cover the full range of QoL score in the population. In this case, the mean of the item difficulty should be close to the mean of QoL score of the participants and greater difference in the means leads to poorer targeting [36].

## Results

### CTT analysis

Table 1 shows the Cronbach's alpha coefficients, means and SDs of each domain of the PedsQL^TM ^4.0 for child self-report and parent proxy-report, and also the results of convergent and discriminant validity. All the domains have adequate internal consistency (greater than 0.7). Scaling success rates for convergent and discriminant validity were 100% in all domains with the exception of physical health in child self-report. In addition, the CCFA supported the fit of a four-factor model for child self-report, RMSEA = 0.059, NNFI = 0.96, RMR = 0.069, and CFI = 0.97, and parent proxy-report, RMSEA = 0.083, NNFI = 0.95, RMR = 0.08, and CFI = 0.96.

**Table 1:** Cronbach's alpha coefficient, convergent and discriminant validity for the PedsQL^TM ^4.0 Generic Core Scales and score domains for Iranian school children

					Convergent validity		Discriminant validity	
					
	No. items	n	Mean ± SD	α	Range of correlation	Scaling success (percent)	Range of correlation	Scaling success (percent)
**Child self-report**								
Total score	23	938	77.18 ± 13.38*	0.85	-	-	-	-
Physical health	8	938	78.32 ± 15.43*	0.70	0.31-0.67	87 (7/8)	0.10-0.42	96 (23/24)
Emotional functioning	5	938	68.46 ± 21.32	0.70	0.58-0.73	100 (5/5)	0.21-0.37	100 (15/15)
Social functioning	5	938	85.06 ± 17.07*	0.74	0.64-0.68	100 (5/5)	0.19-0.42	100 (15/15)
School functioning	5	938	76.22 ± 18.78*	0.69	0.59-0.71	100 (5/5)	0.14-0.35	100 (15/15)
**Parent proxy-report**								
Total score	23	938	73.49 ± 16.65*	0.89	-	-	-	-
Physical health	8	938	72.48 ± 20.79*	0.81	0.52-0.72	100 (8/8)	0.25-0.46	100 (24/24)
Emotional functioning	5	938	68.62 ± 21.85	0.74	0.63-0.77	100 (5/5)	0.25-0.38	100 (15/15)
Social functioning	5	938	80.03 ± 21.68*	0.79	0.66-0.77	100 (5/5)	0.26-0.50	100 (15/15)
School functioning	5	938	73.47 ± 20.69*	0.70	0.55-0.79	100 (5/5)	0.15-0.45	100 (15/15)

* indicates that the mean difference in QoL scores between the child and proxy-report are statistically significant at 5% level.

### CCFA

The values of fit indices for the one-factor CCFA in Table 2 suggest that the physical health domain in self-report, and the emotional and social functioning domains in both self- and proxy-reports were unidimensional scales. However, the values of RMSEA > 0.1 show that the unidimensionality of the physical health domain in proxy-report and the school functioning domain in both self- and proxy-reports may be tentative [29]. In addition, the results of the CCFA showed that in the physical health domain, the values of modification indices between items 1 and 2 in self-report and items 7 and 8 in proxy-report were 65.5 and 282.2, respectively, while those of the other pairs ranged from 0 to 48 in this domain. The values of modification indices between items 4 and 5 in the school functioning domain were 130.5 and 108.5 for self- and proxy-reports, respectively, whereas other values were in the range of 0 to 74. Given the values of the chi-square statistics in Table 2, these large modification indices may indicate a violation of local independence. However, small modification indices between each pair of items in the emotional and social functioning domains confirmed the local independence assumption.

**Table 2:** Goodness of fit indices for the one-factor CCFA model in four domains of the PedsQL^TM ^4.0 Generic Core Scale

	chi-square	d.f	RMSEA	NNFI	RMR	CFI
**Child self-report**						
Physical health	187.98	20	0.091	0.94	0.077	0.96
Emotional functioning	3.49	5	0.000	1.00	0.013	1.00
Social functioning	40.73	5	0.085	0.98	0.046	0.99
School functioning	138.82	5	0.230	0.77	0.120	0.88
**Parent proxy-report**						
Physical health	272.16	20	0.140	0.93	0.091	0.97
Emotional functioning	12.48	5	0.042	1.00	0.026	1.00
Social functioning	37.08	5	0.090	0.99	0.039	0.99
School functioning	143.54	5	0.240	0.79	0.120	0.90

RMSEA: root mean square error of approximation

NNFI: non-normed fit index

CFI: comparative fit index

RMR: root mean square residual

### IRT analysis

Table 3 shows the Rasch-derived item and person separation indices and reliability for each domain. Although all domains of self- and proxy-reports had high values of item separation index and reliability, person separation index and reliability were all below the accepted level.

**Table 3:** Reliability and separation indices in the PedsQL^TM ^4.0 Generic Core Scale

	Child self-report		Parent proxy-report	
	
	person	item	person	item
Physical health				
Reliability	0.54	0.99	0.61	0.95
Separation	1.07	11.93	1.25	4.53
Emotional functioning				
Reliability	0.62	0.99	0.63	0.99
Separation	1.28	9.49	1.30	8.21
Social functioning				
Reliability	0.23	0.87	0.40	0.94
Separation	0.55	2.54	0.82	4.14
School functioning				
Reliability	0.52	0.98	0.56	0.99
Separation	1.05	6.93	1.12	11.15

### Item statistics

Table 4 represents item difficulty, item fit statistics and category frequency for each item of the PedsQL^TM ^4.0 for self- and proxy-reports. The range of difficulty in child self-report was from -1.01 to 0.71 in which the item 'Hard to take a bath or shower' was the easiest task (90% of school children responded "never" to this item) while the item 'Hard to lift something heavy' was the most difficult (only 31% reported "never" to this item). Item difficulty ranged from -0.68 to 0.43 in parent proxy-report in which the item 'Trouble keeping up with schoolwork' was the most difficult item and 'Miss school - not well' was the least difficult one. The percentages of parents choosing "never" for these items were 66% and 40%, respectively. In addition, infit and outfit MNSQ for all the items were within the accepted range, except for the item 'Hard to take a bath or shower' in both self- and proxy-reports being greater than 1.4.

**Table 4:** Item difficulty, infit and outfit MNSQ statistics for each item of the PedsQL^TM ^4.0 Generic Core Scales and frequency response categories

Scale			Child self-report								Parent proxy-report					
	
				Category frequency (%)								Category frequency (%)				
								
	difficulty	infit	outfit	0	1	2	3	4	difficulty	infit	outfit	0	1	2	3	4
**Physical health**																
1. Hard to walk more than a block	-0.23	1.07	0.98	2	4	14	12	67	-0.18	0.83	0.78	7	6	15	14	55
2. Hard to run	0.18	0.76	0.76	3	7	21	18	51	0.13	0.82	0.82	7	11	20	18	44
3. Hard to do sports or exercises	-0.18	0.86	0.75	3	5	12	16	64	-0.03	0.80	0.77	7	7	18	17	51
4. Hard to lift something heavy	0.71	0.99	1.03	8	11	29	21	31	0.23	0.99	1.04	7	9	26	22	36
5. Hard to take a bath or shower	-1.01	2.01	1.63	4	1	2	3	90	-0.04	1.60	1.32	20	2	6	5	67
6. Hard to do chores around house	0.40	1.26	1.22	8	7	20	20	45	0.20	1.00	1.01	9	10	20	20	41
7. Hurt or ache	0.13	0.95	0.95	3	4	23	21	50	-0.09	0.94	1.06	3	7	23	19	48
8. Low energy	-0.01	0.92	0.86	2	6	15	21	56	-0.21	1.11	1.11	2	8	17	20	52
**Emotional functioning**																
1. Feel afraid or scared	-0.30	1.01	1.0	4	4	25	23	44	-0.37	1.05	1.06	3	5	22	23	47
2. Feel sad or blue	-0.16	0.79	0.8	5	6	26	23	41	-0.14	0.71	0.72	4	7	25	24	40
3. Feel angry	0.29	0.78	0.78	8	12	29	22	28	0.37	0.84	0.86	7	15	29	21	29
4. Trouble sleeping	-0.34	1.34	1.24	9	7	12	17	55	-0.23	1.26	1.21	8	7	17	18	51
5. Worry about what will happen	0.52	1.16	1.15	17	12	25	16	30	0.38	1.19	1.14	12	13	22	17	35
**Social functioning**																
1. Trouble getting along with peers	0.16	1.11	1.06	3	4	11	20	62	0.36	1.1	1.06	8	5	14	18	53
2. Other kids not wanting to be friends	-0.20	0.87	0.8	2	3	9	17	70	-0.14	0.84	0.79	4	5	8	19	63
3. Teased	0.11	1.06	1.04	2	3	15	17	63	-0.04	1.1	1.09	4	5	12	20	59
4. Doing things other peers do	0.01	0.97	0.9	3	3	11	17	66	-0.09	0.88	0.87	5	3	12	18	62
5. Hard to keep up when play with others	-0.08	1.03	1.0	2	3	12	16	67	-0.09	1.11	1.08	4	5	12	19	61
**School functioning**																
1. Hard to concentrate	0.16	0.98	0.95	5	7	22	23	43	0.33	0.97	0.93	10	9	20	22	38
2. Forget things	0.32	0.91	0.92	4	7	28	28	33	0.24	0.97	1.01	5	9	26	26	35
3. Trouble keeping up with schoolwork	0.18	0.95	0.9	7	7	20	23	44	0.43	0.94	0.9	13	10	20	17	40
4. Miss school - not well	-0.39	1.08	1.03	3	4	14	19	60	-0.68	1.12	1.02	4	3	9	18	66
5. Miss school - doctor appointment	-0.27	1.19	1.15	4	5	16	18	58	-0.32	1.07	1.15	5	3	16	19	57

0 = Almost always, 1 = Often, 2 = Sometimes, 3 = Almost never, 4 = Never

**Figure 1:**
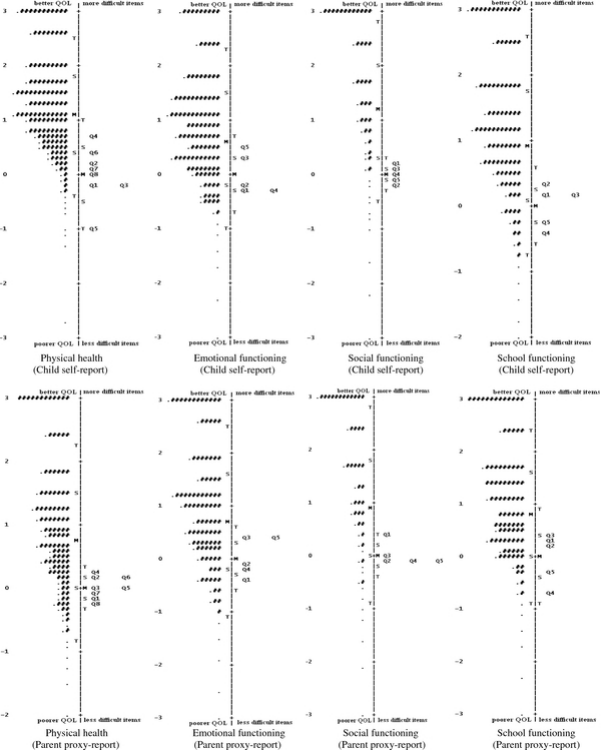
**Person-item map for each domain of the PedsQL^TM ^4.0 Generic Core Scales**.

The person-item map showing the distribution of the children QoL score (left side) and the item difficulty (right side) for each domain of the PedsQL^TM ^4.0 are illustrated in Figure 1. School children with higher QoL score and items with more difficulty were located at the top of the map. Optimal targeting was not observed, since for each domain, the means of the items and persons were fairly far from each other. In all domains, particularly the social functioning domain for both self- and proxy-reports, the majority of school children with the higher QoL score found no corresponding items suggesting that the school children in our sample had a higher QoL score than the average difficulty of the PedsQL^TM ^4.0 items, and they could not be well targeted by the items.

### Rating scale diagnostics

**Figure 2:**
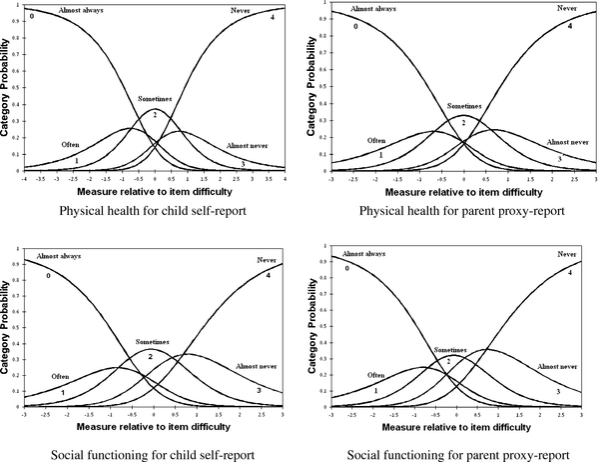
**Category probability curves of five response categories of the PedsQL^TM ^4.0 Generic Core Scales on the physical health and the social functioning domains**.

Table 5 shows that the average measure increased monotonically across rating scale categories for all domains of self- and proxy-reports; moreover, infit and outfit MNSQ statistics were acceptable for all categories except category 0 (almost always) in physical health domain for self-report. However, the step measures did not function as expected and were disordered. Step disordering could be observed in the corresponding category probability curves in Figure 2. For instance, in physical health domain, the intersection of categories 1 (often) and 2 (sometimes) were located on the left side of that of categories 0 (almost always) and 1 (often), and also categories 3 (almost never) and 4 (never) crossed each other before categories 2 (sometimes) and 3 (almost never) did.

**Table 5:** Category functioning statistics for domains of the PedsQL^TM ^4.0 Generic Core Scales

		Child self-report				Parent proxy-report		
	
	Average measure	Infit MNSQ	Outfit MNSQ	Step measure	Average measure	Infit MNSQ	Outfit MNSQ	Step measure
Physical health								
0	0.04	1.25	1.61	None	-0.17	1.05	1.06	None
1	0.24	1.02	1.01	-0.25	0.07	0.99	0.98	-0.05
2	0.54	0.91	0.82	-0.72	0.36	0.96	0.87	-0.67
3	0.91	0.98	0.84	0.81	0.75	0.97	1.01	0.67
4	1.52	0.99	1.00	0.16	1.15	1.02	1.02	0.10
Emotional functioning								
0	-0.51	1.05	1.05	None	-0.47	1.20	1.29	None
1	-0.12	0.86	0.79	-0.34	-0.24	0.80	0.77	-0.70
2	0.33	0.98	0.95	-0.94	0.33	0.94	0.89	-0.79
3	0.82	0.85	0.91	0.70	0.91	0.77	0.86	0.70
4	1.24	1.10	1.10	0.58	1.38	1.10	1.08	0.79
Social functioning								
0	-0.13	1.11	1.11	None	-0.36	1.10	1.12	None
1	0.13	0.89	0.82	-0.30	-0.13	0.76	0.65	-0.23
2	0.59	0.91	0.77	-0.82	0.40	0.94	0.84	-0.68
3	1.17	1.00	1.12	0.43	1.00	0.85	1.01	0.20
4	1.57	1.07	1.04	0.68	1.46	1.16	1.11	0.71
School functioning								
0	-0.24	1.14	1.17	None	-0.31	1.16	1.22	None
1	0.06	0.98	0.94	-0.44	-0.14	0.78	0.70	-0.22
2	0.44	0.91	0.87	-0.91	0.31	0.99	0.99	-0.83
3	0.99	0.96	1.00	0.61	0.86	0.90	1.00	0.45
4	1.45	1.02	1.02	0.74	1.36	1.00	1.00	0.60

0 = Almost always, 1 = Often, 2 = Sometimes, 3 = Almost never, 4 = Never

To evaluate the impact of violation of local independence and unidimensionality assumptions, the RSM for the physical health and the school functioning domains was fitted three times, once including all items in the model and once eliminating each item from the locally dependent pair. For example, school functioning domain was modeled once with all 5 items, without item 4 and without item 5. Based on our findings, extremely trivial differences were found in the parameter estimates and fit of the model.

## Discussion

In this study, it was important to determine whether the PedsQL^TM ^4.0 measures the construct which was intended to measure. We applied the RSM, an IRT model, to reassess the psychometric properties of the Persian version of the PedsQLb^TM ^4.0, which have been previously evaluated using CTT methods [26]. In agreement with previous research [26], CTT methods showed that the Persian version of the PedsQL^TM ^4.0 in southern Iran has an acceptable internal consistency as well as excellent convergent and discriminant validity. Moreover, similar to the original US English version, confirmatory factor analysis indicated that the instrument includes four underlying factors. Although CTT methods produced satisfactory results, the RSM revealed that the validity of the Persian version of the PedsQL^TM ^4.0 should be interpreted with caution. The Rasch RSM showed that in the Persian version of the PedsQL^TM ^4.0 successive response categories for all domains were not located in the expected direction. Although the average measures for all response categories increased monotonically and the five response categories showed acceptable infit and outfit statistics, step measures did not increase monotonically across category responses. These findings are not in the same line with those of the Korean version of the PedsQL^TM ^4.0 [23], which found that the step measures of adjacent response categories increased monotonically and in the expected order except for the social functioning. We do not know exactly whether this problem in our study is a function of the meaning of the response choices in the Persian language or an artifact of a mostly healthy population that did not use the full range of the response categories. Hence, the behavior of the response categories should be evaluated for appropriate functioning in further validation studies, especially in a sample that includes a large number of chronically ill patients.

A review of the published pediatric Patient Report Outcomes Measurement Information System (PROMIS) studies showed that none of them discussed the optimal number of response categories [9,11,14,37-40]. However, it can be inferred from the results that the response category functioning was not problematic in any of these studies except one [9]. It should be noted that most of the pediatric PROMIS studies used the same response scale categories as the PedsQL^TM ^4.0 ("Never" to "Almost Always"). Also, the IRT method used in the current research was different from those in the PROMIS studies; in those studies the GRM had been selected to evaluate the item properties. Unlike the RSM, the number of response categories in the GRM is free to vary across items, and item discrimination parameters are not constant. Moreover, while the among-category threshold parameters in the GRM must be ordered, this property is not a requirement in the RSM [4,5]. Therefore, based on the GRM, in order to test whether the response categories behave well, the threshold parameters should be widely spread out over the trait range [4,5]. It seems that, as compared with parsimonious models, like the PCM or the RSM, less constrained models, including the GRM and the GPCM, provide a more accurate description of the data [5,41]. There are a number of simulation and real-data studies comparing software programs that estimate parameters for polytomous IRT models [42-47]. Generally speaking, because each IRT model has its own function to describe the probability of choosing the response category, item and category parameters cannot be compared directly among the IRT models [48]. According to Linacre [33], when the distance between step measures is less than 1 logits, redefining or combining the categories is required. Step measures in Table 5 suggest that categories 1 (often) and 2 (sometimes) should be combined in all domains for self- and proxy-reports. Moreover, in child self-report, the categories 3 (almost never) and 4 (never) should be combined in physical health and emotional functioning domains.

After this modification, average measures and step measures increased monotonically and no misfitting category was observed. Collapsing categories led to an increase in the range of difficulty, which spanned from -1.33 to 1.77 and from -1.13 to 0.79 for self- and proxy-reports, respectively. However, no improvement occurred in the person-item maps, and the difference in the means of items and persons increased. The effect of varying the number of response categories in rating scales has been assessed by Olivares et al. [49]. Collapsing categories will improve the values of fit indices in IRT models [49], reduce the burden on the respondent and save time [35]. Moreover, they demonstrated that convergent and discriminant validity measures were relatively unaffected by the number of response categories [49]. However, this type of modification usually results in loss of information, including sensitivity of the measure. According to Olivares [49], increasing or decreasing the number of response categories is a trade-off between the precision of the instrument and the goodness of fit. For example, when the number of items is large or if the items are highly discriminating but the goodness of fit of the model is questionable, a researcher might consider using fewer response categories. On the other hand, if the number of items is small or when the items show low discrimination but you expect the model to fit well, you should use more response categories to reduce concerns about poor precision of the instrument [49].

In the present study, the Rasch RSM showed that no item was misfitting with exception of item 5 'Hard to take a bath or shower' in the physical health. A high MSNQ (infit) for this item indicates that it is either poorly designed or is not sufficiently related to the rest of the domain [35,50,51]. Similar to the previous studies [23,24], item 5 on the physical health can be omitted from the instrument because nearly 90% of the children and 70% of the parents have responded "never" to having problems with 'taking a bath or shower' (Table 4).

Similar to the Korean version [23], performing the Rasch analysis on the four domains of the Persian version of the PedsQL^TM ^4.0 revealed that these domains suffered from low person reliability and separation, while item reliability and separation were high. One reason for low person separation is that more than 97% of the participants were healthy school children. Hence, this narrow sample was not able to discriminate between children with equal abilities. Researchers believe that adding more items to the domains or collapsing category responses could improve the performance of these indices [35,49].

The violation of unidimensionality and local independence assumptions in school functioning and physical health domains was a limitation in this study. However, some research indicates that IRT model parameter estimation is fairly robust to minor violations of unidimensionality or local dependence, especially if the latent trait dimensions are highly correlated or if secondary dimensions are relatively small [5]. As we mentioned in the results section, removing or retaining the items responsible for violation of unidimensionality and local independence assumptions did not change the parameter estimation or the changes in the parameters were very small in magnitude. Based on these results, we can conclude that the four domains are sufficiently robust to violation of these assumptions. It should be noted that children rated their HRQoL significantly higher than that rated by their parents. This result was contrary to the findings of the previous studies, which found a tendency in parents to report higher QoL in their children than the healthy school children themselves reported. [52-54]. However, it was in the same line with our previous studies on Iranian children with chronic conditions [27,28].

## Conclusion

The Rasch RSM allowed us to draw the important conclusion that the number of response categories should be reduced from five to three in the Persian version of the PedsQL^TM ^4.0. For professionals who use the PedsQL^TM ^4.0 and are concerned about determining the optimal number of response categories, using a repeated measures design is recommended. In this method, the same instrument is administered repeatedly to the same participants, with a different number of response categories each time. It enables the researcher to capture intra-individual effects that are due to changes in the number of response categories [49].

Moreover, the analysis of DIF is needed to test whether the instrument operates equivalently between healthy school children and children with chronic diseases.

## List of abbreviations

HRQoL: Health Related Quality of Life; QoL: Quality of Life; IRT: Item Response Theory; CTT: Classical Test Theory; DIF: Differential Item Functioning; CCFA: Categorical Confirmatory Factor Analysis; RMSEA; Root Mean Square Error of Approximation; NNFI: Non-Normed Fit Index; CFI: Comparative Fit Index; RMR: Root Mean square Residual; RSM: Rating Scale Model; GRM: Graded Response Model; PCM: partial credit model; GPCM: generalized partial credit model; PROMIS: Patient Report Outcomes Measurement Information System.

## Competing interests

The authors declare that they have no competing interests.

## Authors' contributions

PJ researched the data, wrote the manuscript and analyzed the data, ZB wrote the manuscript and analyzed the data, SMTA supervised the study, and ZS researched the data.
